# Direct-Continuous Preparation of Nanostructured Titania-Silica Using Surfactant-Free Non-Scaffold Rice Starch Template

**DOI:** 10.3390/nano8070514

**Published:** 2018-07-10

**Authors:** Juan Matmin, Irwan Affendi, Salasiah Endud

**Affiliations:** 1 Centre of Foundation Studies UiTM, Universiti Teknologi MARA (UiTM), Cawangan Selangor, Kampus Dengkil, Dengkil 43800, Selangor, Malaysia; affendi7848@puncakalam.uitm.edu.my; 2 Chemistry Department, Faculty of Applied Sciences, Universiti Teknologi MARA, Shah Alam 40450, Selangor, Malaysia; 3 Chemistry Department, Faculty of Science, Universiti Teknologi Malaysia (UTM), Skudai 81310, Johor, Malaysia; salasiah@kimia.fs.utm.my

**Keywords:** nanostructured silica, surfactant-free, non-scaffold template, direct-continuous preparation, sol-gel synthesis

## Abstract

The conventional synthesis route of nanostructured titania-silica (Ti-SiNS) based on sol-gel requires the use of a surfactant-type template that suffers from hazardous risks, environmental concerns, and a tedious stepwise process. Alternatively, biomaterials have been introduced as an indirect template, but still required for pre-suspended scaffold structures, which hinder their practical application. Herein, we report an easy and industrially viable direct-continuous strategy for the preparation of Ti-SiNS from nanostructured-silica (SiNS) using a hydrolyzed rice starch template. This strategy fits into the conventional industrial process flow, as it allows starch to be used directly in time-effective and less complicated steps, with the potential to upscale. The formation of Ti-SiNS is mainly attributed to Ti attachment in the SiNS frameworks after the polycondensation of the sol-gel composition under acidic-media. The SiNS had pseudo-spherical morphology (nanoparticles with the size of 13 to 22 nm), short order crystal structure (amorphous) and high surface area (538.74 m^2^·g^−1^). The functionalized SiNS into Ti-SiNS delivered considerable catalytic activity for epoxidation of 1-naphtol into 1,4-naphthoquinone. The described direct-continuous preparation shows great promise for a cheap, green, and efficient synthesis of Ti-SiNS for advanced applications.

## 1. Introduction

With more than 1000 nanomaterials synthesized corresponding to over 850 templates used in the year 2017, [1,2,3] nanostructured materials are one of unique class of structures that have led to the enhancement of properties and the discovery of new phenomena that are not available for any dense or nonporous materials [4,5]. Of the typical nanostructured materials, nanostructured silica (SiNS) and its functionalized titania-silica (Ti-SiNS) are the most important for drug delivery, gene delivery, biosensors, composite coating, molecular separation, adsorption, catalysis, optoelectronic, medical applications, and other industrial functions [6,7,8]. As a general rule, SiNS and Ti-SiNS can only be obtained by sol-gel synthesis using structure-directing agents (SDAs) of conventional templates [9], which require the formation of micelles (e.g., for MCM-41, SBA-15, FSM-16, and HMS syntheses) [10] or clusters (e.g., for MSA and ERS-8 syntheses) [11,12]. However, most of the available SDAs are produced from non-renewable resources in the industries, namely sodium dodecyl sulfate (SDS), cetyltrimethylammonium bromide (CTABr), Brij-type series (CxEOy), Triton-X 100 (TX-100), and poly(ethylene glycol) of pluronic series (P123, P124) [13,14,15]. The surfactant-type templates can only be used from stepwise synthetic series that require extremely high temperatures with massive energy consumption for industrial production; furthermore, they are environmentally toxic. These raise dire concerns for environmental sustainability and green synthesis issues. In comparison, rice starch is a green, safe, and abundant renewable resource, with a global production of 715 million tons [16]; in contrast, a staggering 89 million tons are wasted annually [17] without being regenerated for useful purposes. No new class of template from green, safe, and renewable resources for industrially viable, low-cost, and sustainable methods in the synthesis of SiNS and Ti-SiNS has been introduced in the last 18 years.

To the best of our knowledge, there are only limited reports on the sol-gel synthesis of SiNS and Ti-SiNS from surfactant-free templates using polymer-silica blends from biomaterials. In 2002, Zhang et al. demonstrated the use of hydrogel with the assistance of surfactant-assisted cetyltrimethylammonium chloride (CTAC) atlow temperatures [18]. Briefly, the authors explained that developing SiNS from a template of biomaterials such as starch involved preliminary work on aging the gel networks into the scaffold template either by an alcogel formation, overnight freezing, salt promotion, and miniaturization or solvent-exchange process, before they can be utilized [19,20,21]. Alternatively, Chao and his co-workers reported extensive work on facilitating porous silica by using an alcogel of starch that requires overnight gelation [22]. Such work follows the traditional polymer-silica blends method of suspending gel networks at less than 5 °C, to be used as scaffold templates for the preparation of SiNS. The major drawback of this method is that the pore structures of the SiNS are irregular, as inherited by the randomly organized gel networks, having hierarchical porosity, and the presence of unnecessary macro-sized large voids that limited the silica’s functionality. More importantly, the current, pre-suspended scaffold template from biomaterials can only synthesize hierarchical porous networks, which eventually limit the materials’ selectivity and functionality [23,24,25]. Evidently, developing simple and effective approaches to synthesizing SiNS without the presence of conventional SDAs and traditional scaffold templates remains a challenge.

This study presents a green, cheap, and efficient strategy for the preparation of Ti-SiNS via simple, direct-continuous sol-gel synthesis of SiNS using rice starch as a template, which is expected to be a promising alternative to SDAs and scaffold templates. Under an acidic medium, insoluble rice starch gel was partially hydrolyzed in water to produce hydrolyzed rice starch, which can be used directly in the sol-gel composition for SiNS, before finally attaching with Ti to produce Ti-SiNS. The morphology, crystal structure, and chemical composition of the prepared Ti-SiNS were characterized. This method offers a faster way (less than 34 h), in terms of synthesis time, to prepare Ti-SiNS, when compared to conventional SDAs (more than 48 h) [15] and scaffold templates (more than 45 h) [16]. The direct-continues strategy enables preparation of Ti-SiNS in four direct schemes that can fit into the conventional process flow used in industrial processes with starch as its starting material. The tedious stepwise-synthesis method that requires synthesis, separation, and purification in each stage before proceeding to other multiple successive steps could be avoided by using a direct-continuous method. Such complexity prolonged the stepwise-synthesis time, and is only convenient for lab scale synthesis, i.e., it is not industrially viable. Moreover, the direct-continuous strategy not only demonstrates the alternative usage of rice starch as a readily available renewable resource template, but also shows that the obtained SiNS were enhanced as the support for the catalyst material. The advantages of this method include short preparation time, inexpensive starting materials (starches and water), environmental soundness, and the simplicity of the process, with lower energy consumption, as well as large-scale production capability, which eventually could shed light on a viable method for food waste management. The SiNS and Ti-SiNS with nano-sized particles and large surface areas are of particular importance in many aspects of human life, especially in the application of advanced materials, in particular, as a good catalyst for the oxidation of 1-naphtol.

## 2. Materials and Methods

**Figure 1:**
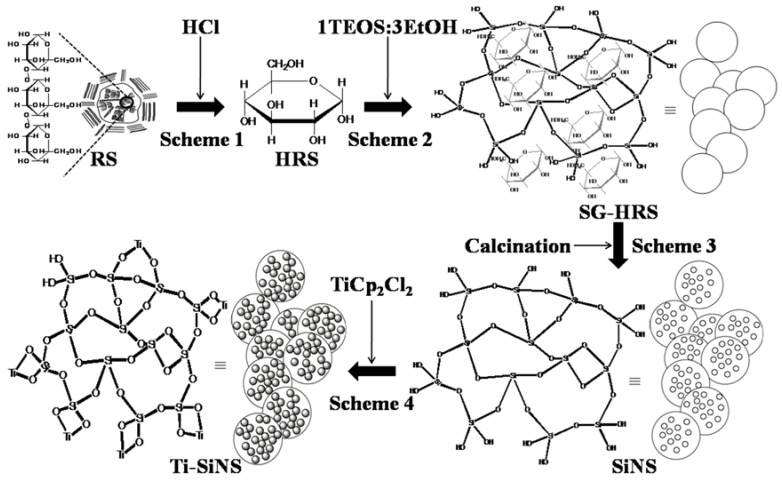
Schemes (**1**–**4**) for direct-continuous preparation of nanostructures titania-silica (Ti-SiNS).

The primary goal of this essay was to prepare Ti-SiNS by direct-continuous hydrolysis of rice starch as a template from a sol-gel synthesis of SiNS. To fulfill that aim, sequential reactions that consisted of four continuous schemes were designed, as illustrated in Figure 1. Hereafter, the starting precursor of rice starch and hydrolyzed starch are denoted as rice starch (RS) and hydrolysis of rice starch (HRS), respectively (Scheme 1). Subsequently, in Scheme 2, aqueous ethanol (EtOH) and tetraethyl orthosilicate (TEOS) were added to the HRS for complete polycondensation of TEOS into a sol-gel paste, which is referred to as SG-HRS. In Scheme 3, the SG-HRS was calcined to remove its organic components to produce SiNS powder. Finally, SiNS was introduced with different titanium (Ti) loadings at 0.5, 1, 2, and 3 wt % to produce (0.5)Ti-SiNS, (1)Ti-SiNS, (2)Ti-SiNS, and (3)Ti-SiNS, respectively in Scheme 4.

### 2.1. Preparation of Nanostructured-Silica (SiNS) and Nanostructured Titania-Silica (Ti-SiNS)

All reagents were analytical grade, and were purchased and used as received, i.e., without further purification. Typically, 20 mmoL of RS (99.9%, Euramco (M) Ptd Ltd., Johor, Malaysia) was mixed with 60 mL of deionized water in a polytetrafluoroethylene (PTFE) bottle to form a gel. To hydrolyze the RS-gel, 2 M HCl was added and heated at 80 °C under constant stirring for 2 h to yield HRS. TEOS was added to a calculated sol-gel composition of TEOS:H_2_O:HCl:CH_3_CH_2_OH at 1:4:0.01:3, in the presence of HRS (38 wt %) at 60 °C for 6 h to produce SG-HRS. The SG-HRS was then calcined at 550 °C to give opal-white colored of SiNS. For Ti-SINS, Ti loadings were introduced to SiNS at different wt % of 0.5, 1, 3, and 5. A weighed amount of titanocene dichloride (TiCp_2_Cl_2_) was first mixed with 50 mL of chloroform in a round bottom flask, before 1 g of SiNS was added. Three milliliters of triethylamine was added to the mixture under constant stirring at 50 °C for 2 h and dried at 130 °C for 24 h to produce Ti-SiNS.

### 2.2. Characterization

Functional groups were determined by a Fourier transform infrared (FTIR) spectrometer (Perkin-Elmer, Spectrum One, Waltham, MA, USA) with a scan wavenumber region of 4000 to 450 cm^−1^, proton (^1^H-) and carbon nuclear magnetic resonance (^13^C-NMR) from Bruker DPX-400 MHz (Rheinstetten, Germany) nuclear magnetic resonance (NMR) spectrometer were used to identify the corresponding chemical shifts. For ^1^H- (at 400 MHz) and ^13^C-NMR (at 100 MHz), chemical shift signals of high resolution-magic angle spinning (HR-MAS) for liquid samples were obtained under 90-free-induction decay (90-FID, Rheinstetten, Germany) pulse sequences with the respect to D_2_O (at 4.5–4.7 ppm) and CD_4_O (49.2 ppm) as standards, respectively. The nitrogen adsorption-desorption measurement was performed using an AUTOSORB-1 Quantachrome volumetric adsorption analyzer (Boynton Beach, Florida, USA) with nitrogen as the adsorbate at 77.35 K for full-scale adsorption-desorption isotherms. The samples were degassed at 363 K for 3 h and held at 433 K for 12 h before analysis. A Barrett-Emmett-Teller (BET) model [23,24,25] was used to calculate the specific surface area, and a Barrett-Joyner-Halenda (BJH) model [23,24] was used to calculate the pore volume distribution and the average pore size. The phase composition was characterized by X-ray diffraction (XRD) equipment on a Bruker D8 Advance instrument (Karlsruhe, Germany) using Cu K*α* radiation (*λ* = 1.54 Å, kV = 40, mA = 40). The morphology of particles was observed using field emission scanning electron microscopy (FESEM) model JEOLJSM-6701F (Peabody, Massachusetts, USA) attached with energy dispersive X-ray (FESEM-EDX, Peabody, Massachusetts, USA), operated at 3.0 kV. Transmission electron microscopy (TEM) micrographs of the samples were recorded by JEM-2100 Electron Microscope JEOL (Peabody, Massachusetts, USA) at 160 kV accelerated voltage. Micrograph images were captured using computerized autofocus system for optimum response of signal-to-noise measurements based on charge coupled device (CCD) camera (Peabody, MA, USA). The thermogravimetric analysis (TGA,) was determined using a thermogravimetric analysis-differential thermal analysis (TGA-DTA) 851 Thermal analyzer (Columbus, OH, USA), with a heating rate of 10 °C min^−1^ from 50 to 1200 °C, under the protection of argon gas. The diffuse reflectance (DR) UV-Vis spectrum was obtained using Perkin-Elmer Lambda 900 ultraviolet-visible/near infrared (UV-Vis /NIR, Waltham, MA, USA) in the wavelength range from 200 to 600 nm and Kubelka-Munk axis, using polytetrafluoroethylene polymer as a standard background.

### 2.3. Catalytic Experiment

The reactions were conducted under atmospheric pressure in a 50 mL double-necked round bottom flask as follows: 1.5 g of 1-naphtol was mixed with equimolar of hydrogen peroxide (H_2_O_2_) in diluted acetonitrile and magnetically stirred under nitrogen gas flow; the freshly activated Ti-SNS (0.25 g) was added and allowed to heat to 70 °C; and samples were collected and analyzed by GC (Agilent Technologies, 690N, Santa Clara, CA, USA) equipped with flame ionization detector (FID) using a ThermoFinnigan (Waltham, MA, USA), HP-5m × 0.32 mm × 0.25 μm column on nitrogen gas carrier. For comparison, the same procedure was performed with SiNS and H_2_O_2_ as the only catalyst.

## 3. Results and Discussion

### 3.1. Characterization of Hydrolysis of Rice Starch (HRS) and SG-HRS

**Figure 2:**
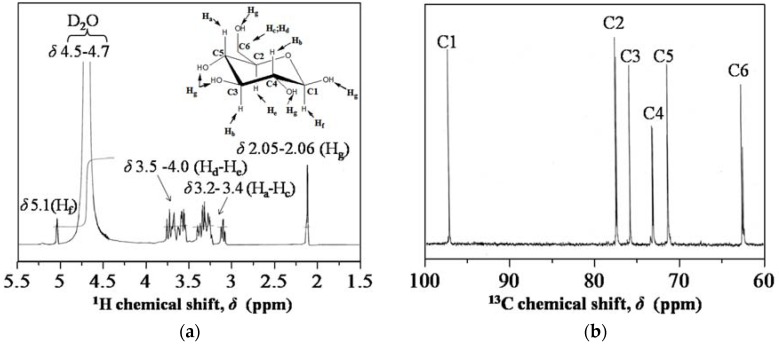
Chemical shifts of hydrolysis of rice starch (HRS) (**a**) proton nuclear magnetic resonance (^1^H-NMR). Inset shows the representative structures of d-glucose; (**b**) carbon nuclear magnetic resonance (^13^C-NMR).

In Scheme 1, the RS powder turned to highly viscous gels upon addingof water. Since water is an important component in the sol-gel composition, the gelatinized starch is, due to granular swelling, the main disadvantage of RS to be readily blended with TEOS for homogeneous sol-gel processes. It is crucial to note that RS is composed of long polysaccharide chains that hydrolyze with water in the presence of acidic media [26,27]. For that reason, the gels were hydrolyzed with HCl to give a dark pale solution in Scheme 1. To confirm the hydrolysis, the dark solution of HRS was unambiguously characterized by ^1^H-, ^13^C-NMR spectroscopy and Benedict Test. For ^1^H-NMR, the following chemical shift at 2.05–2.06 (d, 12H, COCH_3_), 3.2–3.4 (m, 4H, CH_2_CH_2_O), 3.5–4.0 (m, 4H, CHOH), 4.5–4.7 (m, D_2_O solvent), and 5.1 ppm were measured, as indicated in Figure 2a. To illustrate, the corresponding chemical shift at 2.05–2.06 was due to methylene protons of an acetyl group for H_g_. The multiplet chemical shift at 3.3–4 ppm was significant for proton of the anhydroglucose units (H_a_ to H_e_), while singlet chemical shift at 5.1 ppm for H_f_ was significant for equatorial proton from the same unit. For ^13^C-NMR, the chemical shifts at 96.9 (C1), 77.0 (C2), 76.8 (C3), 75.2 (C4), 70.7 (C5), and 61.9 ppm (C6) were measured, as shown in Figure 2b. Both ^1^H- and ^13^C-NMR are in good agreement for chemical shifts of d-glucose. The dark solution of HRS turned to an intense terracotta color on Benedict’s test to confirm the successful hydrolysis of polysaccharide to d-glucose, as shown in Figure S1. This is due to the complex ions of copper (II) citrate, which reacted with terminal carbon at C1 of HRS (mostly d-glucose) to form a red precipitate of Cu_2_O that obeyed a redox reaction. Based on the spectrophotometric determination of Benedict’s solution for HRS [28,29], the maximum d-glucose concentrations of HRS were measured at 0.95 M (in Figure S2).

**Figure 3:**
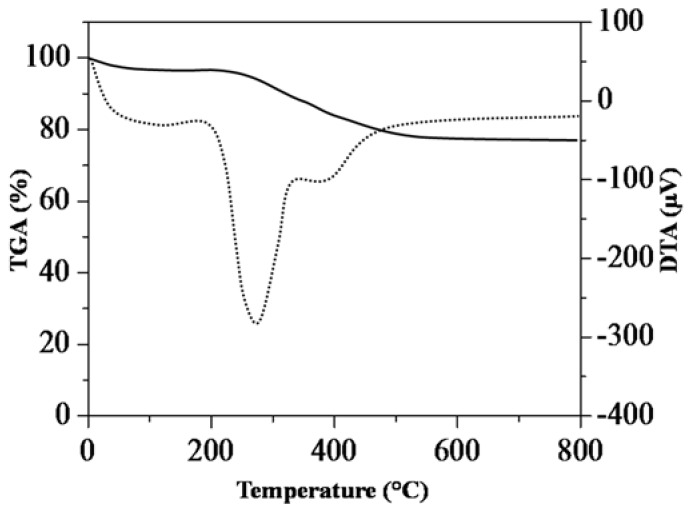
Thermogravimetric analysis-differential thermal analysis (TGA-DTA) thermogram of SG-HRS.

Representative TGA-DTA thermogram on SG-HRS is shown in Figure 3. Based on the thermogram, the initial weight loss (at 8 wt %) was observed from 20 to 150 °C, corresponding to the evaporation of physically adsorbed water (or ethanol) in pores/nano-structures [30,31]. It is worth noting that the breakage, decomposition and oxidative desorption of most organic components usually take place at above 150 °C. In the case of SG-HRS, the major weight loss at more than 10 wt % was measured from 150 to 350 °C, which is associated with weight loss due to the decomposition of organic components, water, and ethanol molecules that are trapped in between the organic/inorganic interface. Finally, the smallest weight loss at less than 2 wt % from 350 to 550 °C corresponds to water evaporation in the internal structures of adjacent silanol (Si–OH) groups. Overall, a total of 20 wt % loss was measured for the SG-HRS sample. This figure is comparable to that observed for typical nanostructured silica/organic composites [32]. Explicitly, the removal of template components in the sol-gel composite by calcinations is a significant step to synthesize SiNS, as indicated in Scheme 3.

### 3.2. Characterization of SiNS

#### 3.2.1. Fourier Transform Infrared (FTIR) Study

**Figure 4:**
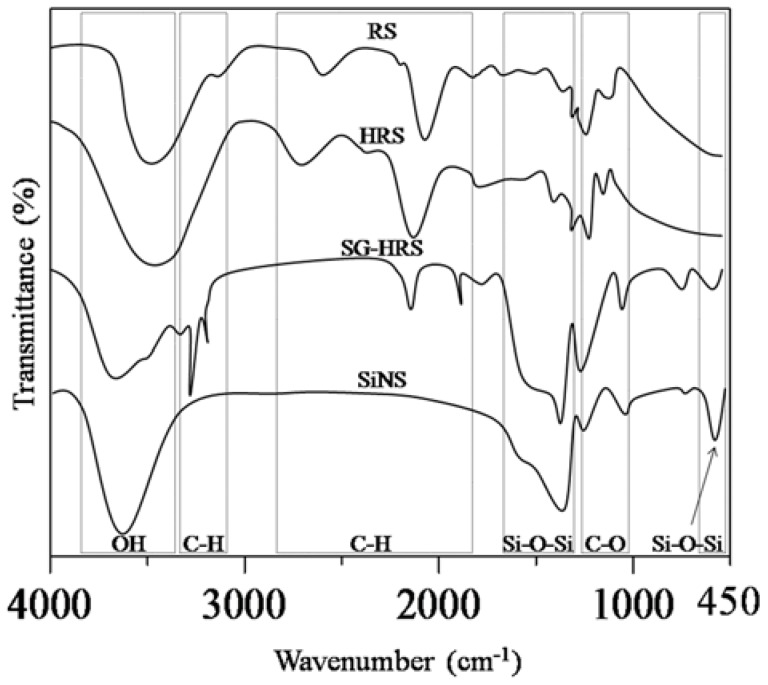
Fourier transform infrared (FTIR) spectra of rice starch (RS), hydrolysis of rice starch (HRS), SG-HRS, and nanostructured silica (SiNS).

The progress of Schemes 1 to 3 from RS to SiNS was closely monitored by FTIR measurements, as shown in Figure 4. In Scheme 1, by comparing both IR spectra of RS and HRS, there was a notable disappearance of C–H stretching at 2860–2970 cm^−1^, indicating a successful cleavage of long polysaccharides chains. Nevertheless, both RS and HRS spectra showed similar vibrational bands of intact glucose monomer. To illustrate, the peaks at 1079 and 1021 cm^−1^ were attributed to the anhydroglucose ring of O–C stretch, whereas vibrational band at 2927 cm^−1^ was assigned to C–H stretches. At around 3400 cm^−1^, there was an extremely broad band due to hydrogen bonded hydroxyl groups (O–H), attributed to vibrational stretches of free, inter-and intra-molecular bound hydroxyl groups. The vibrational bands at 1154, 1079, 1021, and 930 cm^−1^, were attributed to C–O stretching in the fingerprint region. Both RS and HRS spectra showed the presence of water with a vibrational band measured at approximately 1640 cm^−1^, corresponding to the bending mode of adsorbed water. Notably, FTIR spectrum for HRS showed an intense vibrational band at 1670 cm^−1^, corresponding to β-sheet conformation [33].

Notably, the FTIR spectrum for SG-HRS paste showed vibrational bands at 2924, 2854, and 1458 cm^−1^, corresponding to C–H stretch of organic composition in the sol-gel synthesis. The disappearance of these peaks in FTIR spectrum of SiNS indicates that the organic composition is successfully removed. The complete removal of HRS is further supported by the fact that the SG-HRS showed no weight loss upon heating to 800 °C under oxygen in the TGA measurements. Moreover, the adsorbed molecular water in the sample was detected by the presence of a broad peak located between 3350 and 3500 cm^−1^, which corresponds to the overlapping of the O–H stretching band of the hydrogen-bonded water molecule (H–O–H···O–H). Another band that was also attributed to the adsorbed water molecule during the polycondensation process was measured in the region of 1620 to 1640 cm^−1^. In Scheme 3, SiNS showed a typical IR spectrum of silicate (SiO_4_) building unit [34]. The prominent vibrational bands at 1086 and 1230 cm^−1^ were assigned to asymmetric Si–O–Si stretching vibrations. The shift in intensities and band from 1076 cm^−1^ (in SG-HRS) to 1086 cm^−1^ (in SiNS) is due to inter-particle bonding of network strengthening after calcination. Both vibrational bands at 798 cm^−1^ and 464 cm^−1^ are associated with symmetric Si–O–Si stretching having SiO_4_ tetrahedron structures and Si–O–Si bending mode, respectively. Furthermore, the Si–OH stretching of terminal Si–OH groups at the pore walls was measured at 951 cm^−1^.

#### 3.2.2. Electron Microscopy

**Figure 5:**
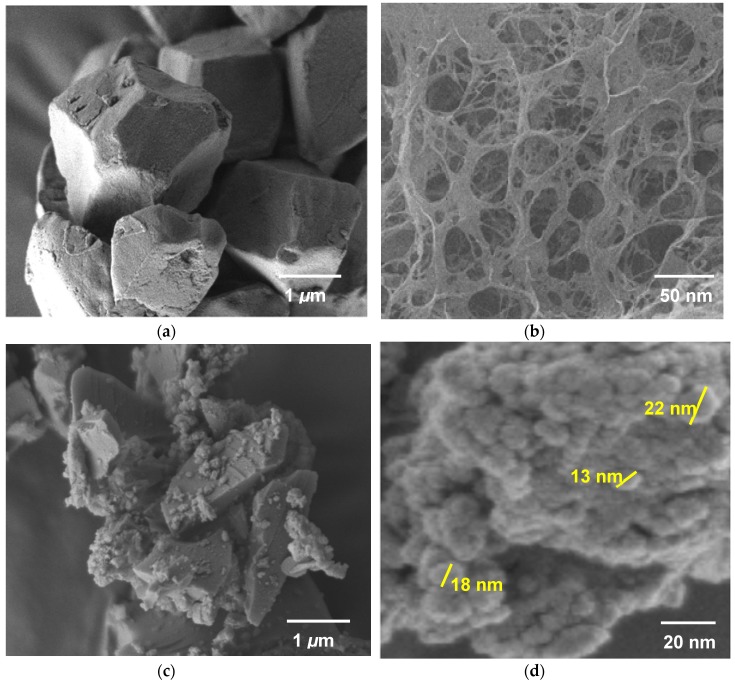
Field emission scanning electron microscopy (FESEM) micrograph of (**a**) RS; (**b**) RS-gel; (**c**) SG-HRS; and (**d**) SiNS.

Figure 5 shows the FESEM micrograph for RS, RS-gel, SG-HRS, and SiNS having polygonal, gelatinized networks, ordered cluster, and pseudo-spherical morphology, respectively. Interestingly, RS changed from its native polygonal shapes in Figure 5a to polymeric networks of RS-gels in Figure 5b through granular swelling in the presence of water [35]. In particular, the RS-gels are composed of entangled fibers with sizes in the range of 20 to 50 nm having interconnected networks. It should also be noted that RS itself is highly resistant tolarge amounts of water, based on evaluated critical gel concentration (CGC) at more than 100 wt %, corresponding to the formation of micro-crystallites of amylose on polymeric gel [36]. On sol-gel composition, the SG-HRS paste showed an ordered cluster with distinct dimensional straight lines of polymer-silica composites, as shown in Figure 5c. Judging from the FESEM micrograph, SG-HRS had an ordered cluster with 3 to 5 micro-sized aggregations that might be inherited from the polygonal shape of RS. Based on FESEM image in Figure 5d, SiNS showed highly aggregated nanoparticles measured in the range of 13 to 22 nm diameters with pseudo-spherical shaped morphology.

**Figure 6:**
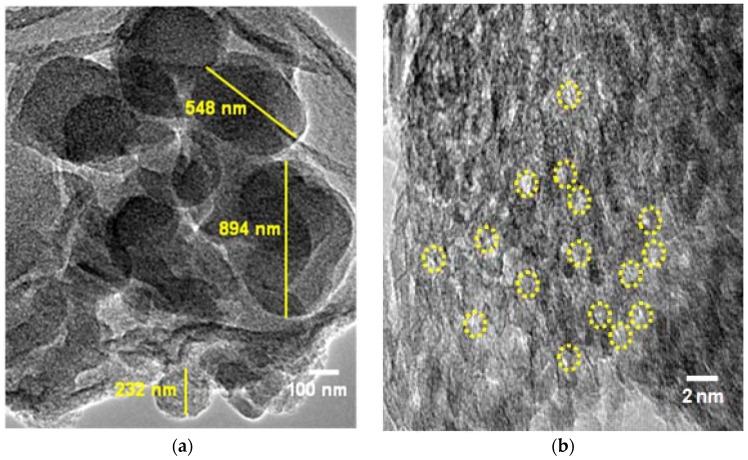
Transmission electron microscopy (TEM) micrograph of (**a**) SG-HRS; and (**b**) SiNS.

The TEM micrograph in Figure 6 shows an ordered cluster and aggregated structures with pores of SG-HRS and SiNS, respectively. For SG-HRS, well-defined clusters with less than 5 micro-sized were measured in Figure 6a. On the other hand, SiNS shows aggregation of microporous structures with almost regular diameters. The present of randomly distributed fine pores were suspected having diameters less than 2 nm as suggested in Figure 6b. Notably, conventional SDAs benefit from its surfactant-type structures to form micelle either in cationic, anionic, or neutral charge, to direct the formation of pores. In the case of SiNS, the adsorption of the oligomeric silica species onto the incipient of HRS, which is rich in d-glucose fiber during the sol-gel transcription, is responsible for the microporosity. It should be noted that the morphology of SiNS undergoes consolidation by the sintering effect on calcination at high temperature, and might also affect SiO_4_ particle uniformity. Another factor that might contribute to the loose aggregation morphology of SiNS are the partial macrophase separation that might be present during the sol-gel aging process.

#### 3.2.3. Physisorption Measurements

**Figure 7:**
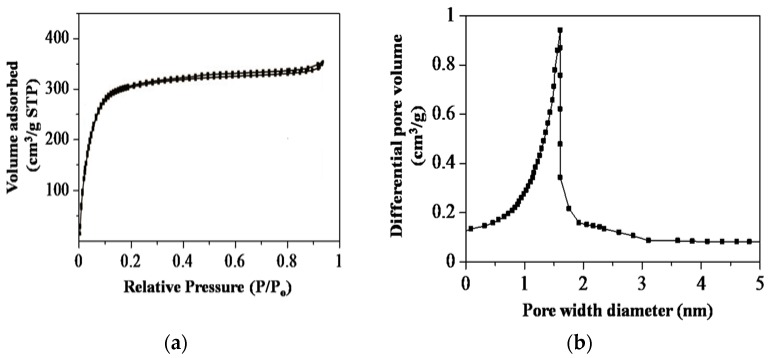
Nitrogen adsorption-desorption measurements (**a**) Isotherms plot; and (**b**) Barrett, Joyner and Halenda method (BJH) models.

Nitrogen adsorption-desorption measurements were used to evaluate the porosity of SiNS, as shown in Figure 7. According to IUPAC classification, the isotherms for SiNS are of a typical type I, based on a well-defined plateau which is significant for highly microporous materials. The primary adsorption occurred at low relative pressure (<0.1), with the absence of a more rounded ‘knee’ indicating that the pore sizes were narrowed, as shown in Figure 7a. The limiting uptake having perfectly reversible isotherm is due to accessible surface pore volume and the presence of a pore size with no obvious pore blocking effect. Adsorption forces (adsorbent to adsorbate) across the pores originated through the filling of these narrow pores. Larger micropores are filled by cooperative effects of adsorbent to adsorbate interactions in the relative pressure region between *P*/*P*_0_ 0.01 ± 0.2. Furthermore, the Brunauer-Emmett-Teller (BET) surface area for SiNS has been experimentally determined to be 538.74 m^2^·g^−1^. The specific surface area obtained is considerably higher than most natural porous materials such as clay (10 to 100 m^2^·g^−1^) and activated graphite (<200 m^2^·g^−1^) [37,38]. To compare with other synthetic porous silica materials, the surface area obtained from this method is also higher; Wu et al. (127 m^2^·g^−1^), Affandi et al. (152 m^2^·g^−1^), and Wei et al. (455 m^2^·g^−1^) [22,39,40]. The total accessible micropore volume present can be regarded as the adsorption space; the process by which this occurs is due to the micropore filling, in accordance to IUPAC definition, which is distinct from surface coverage, and which takes place on the wall of open macropore or mesopore. It is worthy of note that different plots such as Barrett, Joyner and Halenda method (BJH), the Micropore (MP) Method, Density Functional Theory (DFT), Dubinin Plots, and Horvath-Kawazoe (H-K) calculations are widely reported to determine the effective pore size distributions [36,37,38]. In this study, BJH models were calculated in satisfactory fit for SiNS to have an average pore diameter of 1.6 nm, as shown in Figure 7b.

#### 3.2.4. X-ray Diffraction (XRD) Analysis

**Figure 8:**
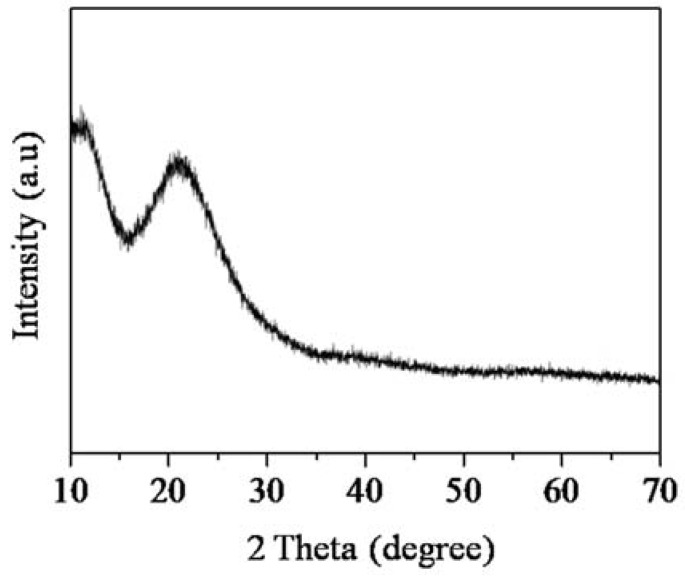
X-ray diffraction (XRD) measurement for SiNS.

XRD is used to identify the structural phase of SiNS, as shown in Figure 8. Based on the diffractogram, SiNS is highly amorphous, as distinguished with a broad peak centered at 2*θ* = 23°, which is in good agreement with JCPDS data (card No. 01-086-1561) [39]. The broad diffraction of the amorphous halo is identical for nanostructured silica with micropores as reported by others [39,40]. From the broad peak, it can be distinguished that SiNS is made up from short order-range of SiO_4_ having tetrahedral atoms with *d*-value of 4 Å that is typical for silica fume and Stoeber particles. The amorphous SiNS are randomly distributed, and do not possess long periodicity, which is likely due to the rapid growth that promoted nucleation of short order SiO_4_ in acidic-media during the sol-gel [41]. More importantly, the lack of distinguishing peaks for SiNS suggested that the template molecules of HRS did not crystallize into long order polymeric chains during the sol-gel process.

Based on the SEM, TEM, nitrogen adsorption-desorption, and XRD analysis, it can be deduced that HRS act as thickener molecules in the sol-gel, and self-assembled into SG-HRS to facilitate the pores formation of 1.6 nm in diameters on the SiNS. Nonetheless, d-glucose was calculated as having 8.0 Å in molecular-size, based on computer-assisted modeling of molecular mechanics-2 (MM2), as shown in Figure S3. Therefore, the proposed mechanism for SiNS should consider the presence of more than one monomer of d-glucose for HRS (in the presence of water) that are adsorbed insilica oligomers onto the cationic surface of the supramolecular aggregates by electrostatic interactions. Subsequently, the silica oligomers are polymerized on the surface of HRS through supramolecular assemblies of low molecular weight gelators (LMWGs) [42] during sol-gel synthesis. The SiNS are obtained after the removal of organic components in the SG-HRS by calcination in air. The homogeneous sol without macroscopic particulation of silicates is formed as a result of the hydrogen bonding between the organic template, in this case, HRS, which is rich in d-glucose, and the intermediate silicate species, Si(OR)_4_-*x*(OH)*x*. This is similar to the scenario where amine surfactants were used as templates [43,44,45]. HRS may also become a facilitating element to the hydrolysis and condensation of the Si–OH groups. It was observed that, at higher concentrations of d-glucose, the sols underwent gelation at a shorter period, indicating that the presence of d-glucose facilitates condensation. In the drying process, the high and increasing viscosity of the system, combined with the affinity of d-glucose for the silicate species, might have contributed to avoiding the inorganic and organic moieties from macroscopic phase separation, as the volatile solvent molecules and reaction by-products (i.e., alcohol and water) gradually evaporated from the system. The silicates and template molecule interactions help in stabilizing the silica framework, and protect it from risks of fracture due to capillary pressure and the increasing internal stress during the drying process. As a result, an organic-inorganic composite which was composed of HRS template and silica with bicontinuous networks in monolithic transparent solids form was obtained. Removing the templates by means of calcination produced silica materials with interconnecting pores.

### 3.3. Characterization of Ti-SiNS

**Figure 9:**
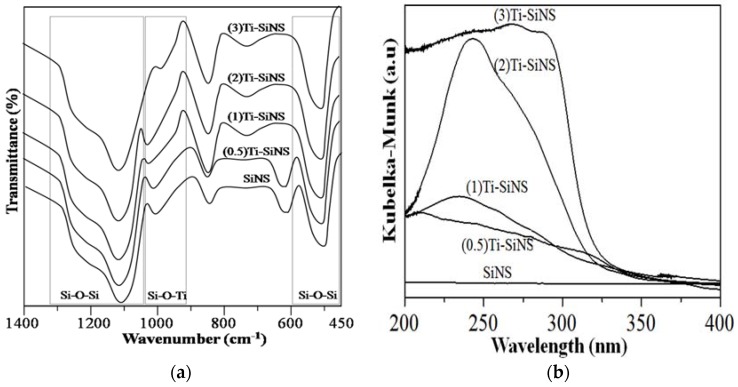
(**a**) FTIR spectra; and (**b**) DR UV-Vis spectra for SiNS, (0.5), (1), (2), and (3)Ti-SiNS.

Figure 9a shows the FTIR spectra for SiNS and Ti-SiNS (1400–450 cm^−1^) with different Ti loadings. Notably, the shift in the measured vibrational band of SiNS at 951 to 960 cm^−1^ for TiSiNS is due to the presence of Ti (IV) on the silica surface. This was made possible by the grafting of Ti via the covalent linkage formed from the hydrolysis of the Ti precursor on the Si–OH. The IR band at 960 cm^−1^ is specific for (1)Ti-SiNS, due to the asymmetric stretching modes of a SiO_4_ unit bonded to a tetrahedral Ti (IV) ion or titanyl [Ti = O] to give Si–O–Ti linkages in the nanostructured frameworks. This band can also be described as the result of superimposition of the Si–OH) stretching mode of the silanol groups with the Si–O–Ti asymmetric stretching mode. To highlight this, all samples showed a similar vibrational band at 798 cm^−1^, that is due to the symmetric stretching/bending of Si–O–Si bridges. As mentioned in the characterization of SiNS, the band at 1087 cm^−1^, and shoulder at 1220 cm^−1^, were assigned to the Si–O–Si asymmetric stretching. Of particular interest, (3)Ti-SiNS gave an almost featureless vibrational band at 949 cm^−1^, indicating pore deficiencies on the interaction of octahedral Ti; this is in agreement with the literature [46,47].

The DR UV-Vis spectra in the range of 400 to 200 nm for SiNS and Ti-SiNS with different Ti loadings are depicted in Figure 9b. For SiNS, the spectrum did not show any significant signal in this region. The maximum absorption band shifted from lower to higher wavelengths, and the intensity increased as the Ti loading increased. To elaborate, absorption bands at less than 230 nm was a result of oxygen to tetrahedral Ti (IV) from ligand to metal charge transfer (LMCT), which can be measured for (0.5)Ti-SiNS and (1)Ti-SiNS. This absorption band is a strong indication of the good distribution of Ti in the silica framework [48]. The (2)Ti-SiNS had a maximum band at 250 nm, and an additional strong shoulder from 260 to 330 nm, suggesting the presence of isolated tetrahedral and octahedral species in the silica lattice, respectively [49]. The samples with higher Ti loading of (3)Ti-SiNS showed even broader absorption bands, and resembled that of TiO_2_ samples of Degussa P25 [50]. Hence, TiO_2_–like clusters should be abundant in this sample. It can therefore be inferred that catalytically active tetrahedral Ti (IV) were highly present in all the Ti-SiNS samples with Ti loading ≤2 wt %. Nevertheless, the additional broad feature in the tetrahedral character within the Ti environment implies an incipient oligomerization of Ti^4+^ species.

In order to confirm the presence of Ti species in Ti-SiNS, all the samples were analyzed by FESEM-EDX. The (1)Ti-SiNS showed an obvious carbon, oxygen, silica, and Ti element measured on the selected area of micrograph, as shown in Table 1. It is worth noting that the presence of carbon as part of the samples was observed, since a carbon tape was used for taking measurements. To represent this point, mapping images on EDX of (1)Ti-SiNS that give a good distribution of Ti is shown in Figure S4. On the other hand, the Ti was distributed uniformly in SiNS, as was expected, and clarified even at the 1 wt % Ti to SiNS in the EDX measurement. The Ti was found to be dispersed in the pores of SiNS.

#### Catalytic Activity

**Figure 10:**
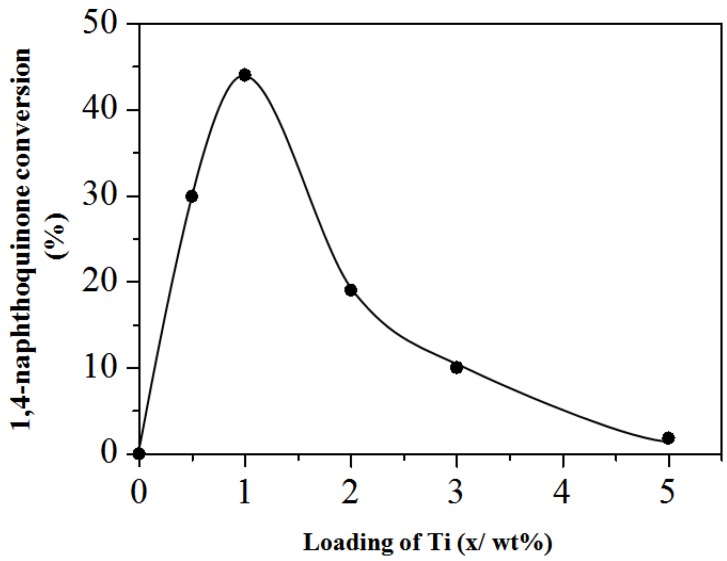
Catalytic performance of Ti-SiNS on oxidation of 1-naphtol to 1,4-naphtoquinone.

To further verify the authenticity of the sample, all of the Ti-SiNS in scheme 4 were tested for oxidation of 1-naphtol to 1,4-naphthoquinone using aqueous H_2_O_2_ for 12 h. The synthesized titania-silica (TiO_2_-SiO_2_) underwent catalyst reactivity evaluation by comparing the conversion percentage of reaction, and selectivity of product, using the catalyst and without the catalyst (control) in the oxidation of 1-naphtol reaction for 1,4-naphthoquinone, using standards equations. Figure 10 shows the percentage of 1,4-naphthoquinone conversion against the amount of Ti loading. For Ti loading at ≤1 wt %, it can be clearly seenthat the catalytic activity of Ti-SiNS increases as the % Ti loadings increases. In the case of (0.5)Ti-SiNS, the 1,4-naphthoquinone was measured at 29.9%. The resultstrongly suggested that 1 wt % gave the highest conversion at 44%. On the other hand, a higher loading of Ti at ≥2 wt % caused a lower catalytic activity. When the Ti loading amount was 2 wt %, the conversion of 1,4-naphthoquinone dropped to only 19%. Unfortunately, the results showed that the excess loadings of titanium (at 3 wt % Ti) caused pore blockages or channel choke, which corresponds to 10% conversion. It is of interest that 5 wt % of Ti-SiNS showed a drastic decrease for conversion of 1-naphtol to 1,4-naphthoquinone at only 1.8%, suggesting pore blockages and defects on catalytic sites. For the controlsample and mixture with SiNS, there was no conversion of 1-naphtol to 1,4-naphthoquinone measured during the catalytic studies. This is mainly because of the absence of initiator that first reacts with H_2_O_2_. These results indicate considerable catalytic activity for epoxidation reaction using Ti functionalized on Si-based catalysts, as compared to Marchese et al. (at 22%) and Oldroyd et al. (at 36%) [48,49].

## 4. Conclusions

In summary, RS can be hydrolyzed in water under acidic conditions to produce HRS (mainly d-glucose) in a sol-gel composition. The HRS was successfully employed as a surfactant-free non-scaffold template in preparing SiNS with a high surface area of (at 538.74 m^2^·g^−1^). The SiNS has pseudo-sphere morphology and amorphous structure that is comparable to conventional nanostructured silica having meso/microporous. The Ti-SiNS delivers considerable catalytic activity, especially for (1)Ti-SiNS (with lower Ti loading at 1 wt %) on epoxidation of 1-naphtol to 1,4-naphthoquinone. It is worth noting that the present method is simple and convenient compared to other existing sol-gel methods, such as SDAs and pre-suspended scaffold template methods. The surface area of SiNS is within the top group of high-area amorphous silicas (>500 m^2^·g^−1^), and is much higher than the majority of industrial amorphous silicas (300–400 m^2^·g^−1^), making it much more competitive, especially in applications involving surface adsorption technologies. Moreover, the pore width of the silica particles is very small at 1.6 nm, which will enable nano-scale surface modifications, including functionalization, to be developed for new types of applications. The fine porosity evident in TEM images is one of the more obvious differences in comparison with the images of other conventional high-area silicas; this indicates an excellent potential for new gas adsorption and gas separation applications. Finally, this paper has proposed a low-cost, efficient, and environmentally-friendly strategy for direct-continuous preparation of Ti-SiNS from SiNS using a surfactant-free non-scaffold HRS template for advanced applications. In the future, these findings might shed light on studies of polysaccharides-based biomaterials as readily available templates for advanced nanomaterials applications.

## Figures and Tables

**Table 1:** Energy dispersive X-ray (EDX) analysis for element composition in (1) nanostructured titania-silica (Ti-SiNS).

Elements	Atomic Percentage
Silica	47.31
Carbon	30.65
Oxygen	19.27
Titanium	2.77

^1^ Si:Ti = 1.00 wt %.

## Supplementary Materials

The following are available online at http://www.mdpi.com/2079-4991/8/7/514/s1, Figure S1. Benedict’s test (**a**) Hydrolysis of rice starch (HRS in dark pale); and (**b**) HRS on Benedict’s solution (Brick’s red). Figure S2. Spectrophotometric determination of Benedict’s solution. Figure S3. Molecular-sized based on computer assisted modeling of molecular mechanics-2 (MM2) modeling of d-glucose. Figure S4. Field emission scanning electron microscopy with energy dispersive X-ray (FESEM-EDX) measurements (**a**) Micrograph of selected image; and (**b**) Energy dispersive X-ray (EDX) spectrum collected from (1)Ti-SiNS.
