# Supplementary material for: Direct-Continuous Preparation of Nanostructured Titania-Silica Using Surfactant-Free Non-Scaffold Rice Starch Template
Source: Nanomaterials (Basel). 2018 Jul 10;8(7):514. doi: 10.3390/nano8070514 (PMC6071009; doi:10.3390/nano8070514)
Supplement: Supplementary file 1 [file nanomaterials-08-00514-s001.pdf]

nanomaterials

MDPI

Supplementary materials

Direct-Continuous Preparation of Nanostructured Titania-Silica using Surfactant-Free Non-Scaffold Rice Starch Template

Juan Matmin[1,2,*], Irwan Affendi[1] and Salasiah Endud[3]

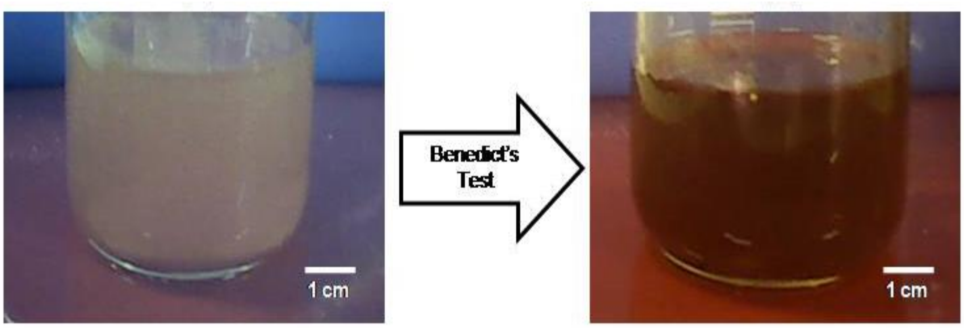

(**a**) (**b**)

Figure S1. Benedict's test (**a**) Hydrolysis of rice starch (HRS in dark pale); and (**b**) HRS on Benedict's solution (Brick's red).

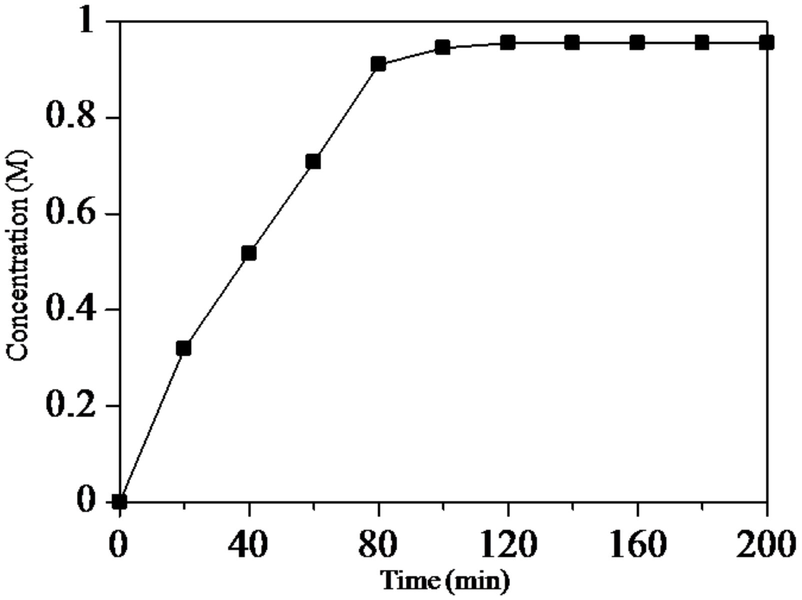

Figure S2. Spectrophotometric determination of Benedict's solution.

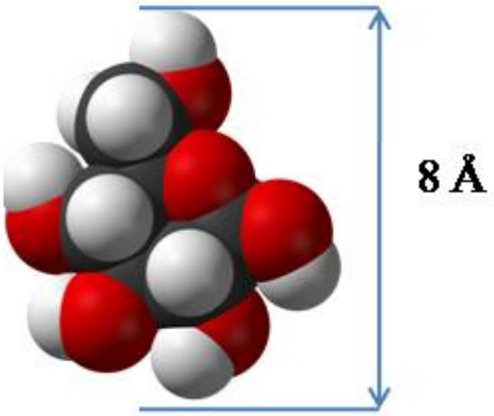

Figure S3. Molecular-sized based on computer assisted modeling of molecular mechanics-2 (MM2) modeling of D-glucose.

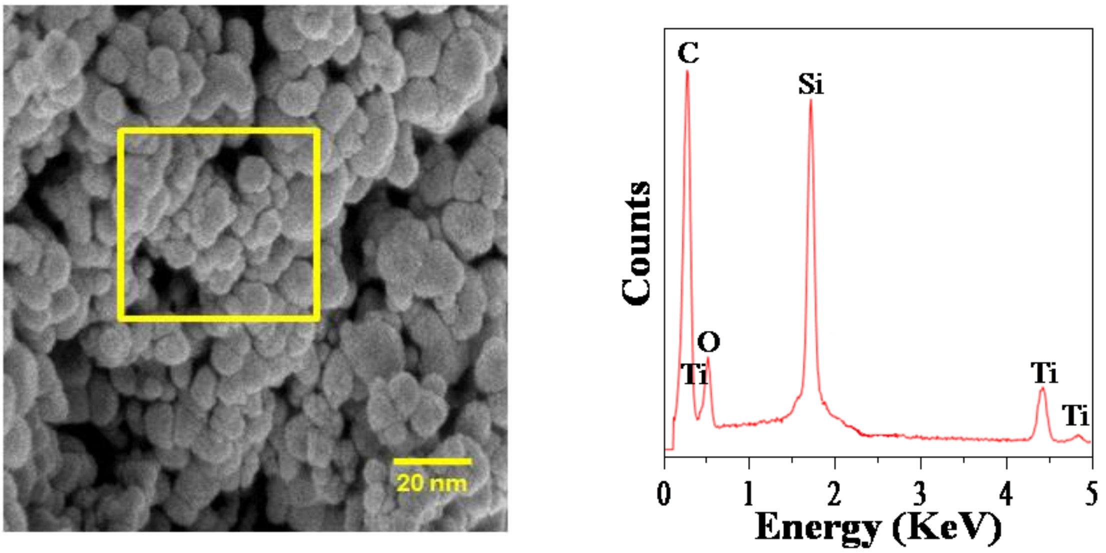

(**a**) (**b**)

Figure S4. Field emission scanning electron microscopy with energy dispersive X-ray (FESEM-EDX) measurements (**a**) Micrograph of selected image; and (**b**) Energy dispersive X-ray (EDX) spectrum collected from (1)Ti-SiNS.
